# Associations between early-life food deprivation during World War II and risk of hypertension and type 2 diabetes at adulthood

**DOI:** 10.1038/s41598-020-62576-w

**Published:** 2020-04-01

**Authors:** Julia Mink, Marie-Christine Boutron-Ruault, Marie-Aline Charles, Olivier Allais, Guy Fagherazzi

**Affiliations:** 1 Sciences Po, department of economics, Paris, 75007 France; 2 Inserm U1018, Center for Research in Epidemiology and Population Health (CESP), Paris-South Paris Saclay University, Villejuif, 94807 France; 3 Inserm U1153, Center of Research in Epidemiology and Statistics, Villejuif, 94807 France; 4 Université Paris-Saclay, INRAE, UR ALISS, Ivry-sur-Seine, 94205 France; 5 Luxembourg Institute of Health (LIH), Department of Population Health, Strassen, 1445 Luxembourg

**Keywords:** Diseases, Risk factors

## Abstract

The Developmental Origins of Health and Disease (DOHaD) framework suggests that early-life experiences affect long-term health outcomes. We tested this hypothesis by estimating the long-run effects of exposure to World War II-related food deprivation during childhood and adolescence on the risk of suffering from hypertension and type 2 diabetes at adulthood for 90,226 women from the French prospective cohort study E3N. We found that the experience of food deprivation during early-life was associated with a higher risk of developing type 2 diabetes (+0.7%, 95% CI: 0.073–1.37%) and hypertension (+2.6%, 95% CI: 0.81–4.45%). Effects were stronger for individuals exposed at younger ages. Exposed individuals also achieved lower levels of education, slept less, and were more frequently smokers than unexposed individuals. These results are compatible with both the latency and the pathway models proposed in the DOHaD framework which theorise the association between early life exposure and adult health through both a direct link and an indirect link where changes in health determinants mediate health outcomes.

## Introduction

Based on the relationship between experience and the biology and psychology of development, the Life Course Health Development (LCHD) and the Developmental Origins of Health and Disease (DOHaD) framework suggest that risk factors, protective factors, and early-life experiences affect individuals’ long-term health and disease outcomes. Although exposure to particular environments and experiences appear to influence health development at all stages, it has been suggested that exposure to environmental insults during childhood and adolescence has particularly powerful and long-lasting consequences on health due to the persistence of bio-behavioural attributes that are acquired early in life^1–4^.

Many studies examined the relationship between early living conditions and health in later life using cohorts exposed to historical events as a natural experiment. Concerning developed countries, several papers have investigated the impact on health of WWII events, particularly the 1944-45 Dutch famine^5–10^, and the 1941-44 Leningrad siege^11,12^, and reported a higher incidence of breast cancer, type 2 diabetes, cardiovascular disease, stroke, and hypertension among cohorts exposed to famine during childhood or adolescence.

However, in most studies the health outcomes were self-reported, thus subject to errors and reporting bias compared with other sources of information, such as medical records or laboratory measurements^13–15^. In addition, many authors were unable to distinguish the direct effects of early life conditions from potential indirect effects mediated through changes in health behaviours and other health determinants, since such information is rarely available. Accounting for such mediating effects appears to be important; indeed, for example, hardship during childhood can have immediate adverse effects on schooling decisions and outcomes^16^, and thus affect health outcomes through reduced achieved education or adult socioeconomic status^17^. The experience of hardship may also influence an individual’s substance consumption or dietary pattern, which could in turn impact health outcomes^18,19^. Finally, many studies did not investigate heterogeneity of effects with respect to age at exposure and are therefore not able to inform policy makers which early life periods are particularly critical for health development.

In this paper, we used data from the French prospective cohort study E3N including ca. 100,000 women born between 1925 and 1950 to investigate the effects of exposure to World War II (WWII)-related under-nutrition during childhood and adolescence on the probability of suffering from hypertension, type 2 diabetes, and obesity later in life while considering potential confounders. In contrast to most existing studies, we used objectively measured health outcomes and also studied the relationship between food-deprivation and subsequent health-affecting behaviours (later-life sleep duration, tobacco consumption, and diet in terms of carbohydrate, lipid, protein and calorie intake) to explore potential indirect effects on health. Finally, we investigated whether the effects are heterogeneous with respect to age at exposure.

After the defeat in the Battle of France (10 May–25 June 1940), the French population experienced almost a decade of severe food supply problems. Access to food was directly affected by the military actions, the fragmentation of the national economy due to the German occupation, the German requisitions, and the contraction of imports and agricultural output^20^. A food rationing system was implemented in September 1940 but did not provide a sufficient diet. The initial rations for adults provided less than 1,700 calories per day and went as low as 1,000 calories in 1944. The most significant losses were in protein and fats. Only consumers with sufficient wealth or with connections to food-producing relatives or friends in the countryside could meet their nutritional needs through purchases on the black market or reception of care packages. Economic recovery required years and the last wartime controls were not lifted until 1949^21^.

The global costs of type 2 diabetes, hypertension, and obesity, and most notably the costs associated with cardiovascular disease as a consequence of these conditions are large and will substantially increase in the coming decades^22–24^. Knowing more about the impact of early childhood conditions on adult health behaviour and health outcomes may allow policy makers to shift away from an emphasis on treatment in the later stages of disease to earlier, more effective preventive strategies and interventions to maximise health development.

## Methods

### Study population

The Etude Epidémiologique auprès de femmes de la Mutuelle Générale de l’Education Nationale or E3N is a French prospective cohort study which was initiated in 1990 to investigate risk factors of cancer and other major non-communicable diseases in women. E3N participants were insured through a national health system that primarily covered teachers and their families, and were enrolled in the study from 1990 onward after they had returned a baseline self-administered questionnaire. The cohort included nearly 100,000 women born between 1925 and 1950. Follow-up questionnaires were sent approximately every 2–3 years and addressed general and lifestyle characteristics together with medical events; the latter especially included cancers, cardiovascular diseases, type 2 diabetes, depression, fractures, and asthma. Our study includes data from the follow-up questionnaires until 2014. The follow-up questionnaire response rate remained stable over the whole study period at approximately 80%^25^. Due to active and passive follow-up (through the transfer of information on addresses, hospitalisations, vital status, reimbursed drugs from the files of the insurer MGEN who insures teachers and their families), the overall loss to follow-up was less than 3%. We used the entire E3N study population for which we have non-missing data on at least one of the health outcomes (diabetes, hypertension, obesity defined as BMI > 30 or overweight defined as BMI > 25) which resulted in a population of 90,226 women. All E3N study participants signed a written informed consent letter and the study protocol was reviewed and approved by the French National Commission for Data Protection and Privacy (ClinicalTrials.gov Identifier: NCT03285230).

### Hypertension and type 2 diabetes assessment

Before 2004, all potential cases of type 2 diabetes were identified through follow-up questionnaires that included questions on the diagnosis of diabetes, diabetes-specific diet, diabetes drugs, and hospitalisations for diabetes. All potential cases were then contacted and asked to answer a diabetes-specific questionnaire that included questions on the circumstances of diagnosis (year of diagnosis, symptoms, biological exams, and fasting or random glucose concentration at diagnosis), diabetes therapy (prescription of anti-diabetic diet or physical activity, list of all used glucose-lowering drugs along time), and the most recent concentrations of fasting glucose and HbA1c. In order to be considered as validated, a potential case must have reported at least one of the following: (1) fasting plasma glucose ≥ 7.0 mmol/l or random glucose ≥ 11.1 mmol/l at diagnosis; (2) use of a glucose-lowering medication; (3) most recent values of fasting glucose or HbA1c concentrations ≥ 7.0 mmol/l or ≥ 7%, in the diabetes-specific questionnaire. After 2004, cases were identified through the drug reimbursement insurance database. The validation algorithm used in the E3N cohort to assess type 2 diabetes cases has been largely accepted and used in several previous publications (e.g. Mancini *et al*.^26^). All women who were reimbursed at least twice for any glucose-lowering medications during one year were considered to be validated cases of diabetes^27^.

Participants were asked to report whether they had hypertension at baseline and in each follow-up questionnaire sent every 2–3 year, the date of diagnosis, and the use of anti-hypertensive treatments. The month and year of diagnosis were provided for most cases (69%). For individuals who were missing the month of diagnosis (14% of cases), it was imputed to June of the year of diagnosis. The median time between the date of diagnosis and the date of response to the first questionnaire after diagnosis was 12 months. Thus, for the cases (n = 17%) with no year of diagnosis, we assigned it to be 12 months before they reported hypertension in a questionnaire. In 2004, a drug reimbursement database became available for 97.6% of participants. We used the self-reported date of diagnosis or the first date of drug reimbursement for anti-hypertensive medications (Anatomical Therapeutic Chemical Classification System codes C02, C03, C07, C08, and C09) whatever happened first, as the date of diagnosis for cases identified after 2004. In addition, using the information of the MGEN health insurance plan drug claim database, we assessed the validity of self-reported hypertension within the E3N cohort. We compared hypertension self-report to anti-hypertensive drug reimbursement (any of the above specified codes). A positive predictive value of 82% was observed among women alive in January 2004 and followed up to their response to the last considered questionnaire in 2008^28^.

To gain statistical power for some of the regression analyses, we grouped the health outcome indicator variables into a new dichotomous variable equal to one if the individual was affected by any of the health conditions and zero otherwise.

### Cofactors assessment

The E3N data provide information on a wide range of individual characteristics. Besides the date and place of birth, educational achievement, weight and height, a measure for the level of stress at work, and marital status, the data included information on early childhood conditions such as preterm birth, birth weight and height, age of the mother and father at birth, indicator variables for the individual’s and her father’s socioprofessional category, whether the individual lived on a farm, number of siblings, and information on the presence of type 2 diabetes and hypertension in the family. The data also included information on health behaviours, such as tobacco smoking, average hours of sleep per night, and dietary intake (used here as carbohydrate, protein, fat, and total calorie intakes).

### Exposure to WWII-related food deprivation during childhood and adolescence

In the first questionnaire, the participants were asked how much they suffered from food deprivation related to WWII. The wording of the question was as follows: “How did you suffer from the food deprivations of the last World War?”. The possible answers were “not born at the time”, “not at all or little suffering”, “moderate suffering”, “a lot (continuous hunger)”, “enormously (deportation)”. Albeit subjective, this variable can be considered as a proxy measure of the intensity of the individual’s experience of hardship during WWII. From this variable, we constructed a dichotomous variable equal to one if the individual claimed to have suffered from hunger at least moderately and zero otherwise.

### Statistical analysis

We regressed the health outcomes (diabetes, hypertension, obesity, overweight) and the health determinants (education, sleep duration, tobacco smoking, and diet in terms of carbohydrate, protein, lipid, and total energy intakes) on exposure to famine and a range of individual and family characteristics using Logit regressions to estimate marginal effects at the mean. The coefficient of the marginal effect at the means gives the change in probability of a health condition occurring for a one unit change in the explanatory variable when the values of all covariates within the sample are set to their means. We tried out different model adjustments to see whether our results were robust to the inclusion of the different cofactors. We included year of birth and place of birth fixed effects in all models, and clustered standard errors at the level of the department. The results were also robust to clustering at the level of the postcode area.

For the regressions on the health outcomes, we also estimated models in which we interacted exposure to famine with dummy variables indicating whether the individual belongs to a certain age group to investigate whether food deprivation impacts health outcomes differently when individuals are exposed at different stages of development. We defined five age cohorts: those born before WWII from 1925–1929 (aged 10–15 years at the beginning of WWII), born 1930–1934 (aged 5–10 years), born 1935–1939 (aged 0–5 years); those born during the war, i.e. 1940–1945, and those born after 1945.

All methods were performed in accordance with the relevant guidelines and regulations.

## Results

### WWII and risk of hypertension and type 2 diabetes at adulthood

Table 1 shows general summary statistics. Table 2 presents the results from our first model in which we estimated differences in the health outcomes for individuals who declared having suffered at least moderately from hunger during WWII and those who declared not having suffered. Our findings suggest that the experience of WWII-related food deprivation during early life is linked to a higher probability of developing type 2 diabetes and hypertension later in life. We did not find evidence for effects on the probability of being obese or overweight. More precisely, individuals who declared having suffered at least moderately from WWII-related hunger during childhood or adolescence had a 0.7% (95% CI: 0.073–1.37%) higher risk of suffering from type 2 diabetes and a 2.6% (95% CI: 0.81–4.45%) higher risk of suffering from hypertension at adulthood compared to unaffected individuals.

**Table 1:** Summary statistics.

	Unexposed (Hunger = 0)				Exposed (Hunger = 1)			
	Mean or %	Min	Max	n	Mean or %	Min	Max	n
***Health outcomes***								
Diabetes (%)	5.89	0	1	69321	9.03	0	1	18712
Hypertension (%)	31.03	0	1	69321	43.91	0	1	18712
Obesity, BMI > 30 (%)	2.69	0	1	68155	4.37	0	1	18291
Overweight, BMI > 25 (%)	15.45	0	1	68155	23.53	0	1	18291
Any health condition* (%)	33.47	0	1	69321	47	0	1	18712
***Health behaviours***								
Sports in childhood (h/week)	41.51	0	215.30	68748	46.19	0	215.30	18555
Hours of sleep (h/day)	7.59	0	21	49614	7.50	3	15	12351
Tobacco usage (%)	34.02	0	1	69173	27.36	0	1	18670
Carb. intake (g/day)	232.93	0	1825.69	53194	228.33	0	1126.00	14029
Protein intake (g/day)	93.09	0	835.77	53194	89.70	0	344.08	14029
Lipids intake (g/day)	90.60	0	917.43	53194	84.43	0	368.27	14029
Calorie intake (g/day)	2198.97	0	20501.27	53194	2107.34	0	7458.53	14029
***Covariates***								
Year of birth	1942	1925	1950	69321	1933	1925	1950	18712
Live with partner (%)	82.69	0	1	67152	76.92	0	1	17949
Higher education (%)	37.51	0	1	67030	28.71	0	1	18015
Born preterm (%)	02.92	0	1	69321	02.75	0	1	18712
Number of siblings	2.20	0	24	45575	2.17	0	20	12050
Age of mother at birth	28.19	13	57	58825	28.13	13	56	15610
Age of father at birth	31.40	10	93	57974	31.24	10	85	15350
Lived on a farm (%)	23.56	0	1	55202	20.62	0	1	14154
Physically stressful job (%)	23.4	0	1	63399	33.87	0	1	16471
Mentally stressful job (%)	82.11	0	1	65216	85.15	0	1	17384
Deprivation index	− 0.26	− 4.11	2.67	50214	− 0.29	− 4.11	2.51	13181
Relative had diabetes (%)	9.45	0	1	69321	7.5	0	1	18712
Relative had hypertension (%)	27.92	0	1	69321	23.93	0	1	18712
Woman of high SES (%)	12.07	0	1	54751	18.59	0	1	14119
Woman of middle SES (%)	85.24	0	1	54751	76.57	0	1	14119
Woman of low SES (%)	2.69	0	1	54751	4.84	0	1	14119
Father of high SES (%)	22.38	0	1	51035	22.75	0	1	13022
Father of middle SES (%)	59.65	0	1	51035	56.4	0	1	13022
Father of low SES (%)	17.96	0	1	51035	20.85	0	1	13022

This table reports the mean value or the proportion (in %), the minimum and maximum values and the number of non-missing observations for each of the variables used in the study separately for unexposed and exposed individuals.

*Any health condition: diabetes and/or hypertension.

**Table 2:** Marginal effect at means of having experienced hunger on different health outcomes.

	Diabetes	Hypertension	Obesity	Overweight
Hunger	0.0072^**^	0.0263^***^	−0.0013	0.0053
	(0.0033)	(0.0093)	(0.0017)	(0.0055)
Num. obs.	26861	26861	26539	26539
Log Likelihood	−5112.12	−16038.27	−2388.31	−9684.18

***p < 0.01, **p < 0.05, *p < 0.1.

The model includes birth-year and department fixed effects, indicator variables for whether the individual lives with a spouse, achieved higher education, has been born preterm, lived on a farm for more than 3 months during childhood and whether the individual or her father is of high socioeconomic status, the age of the individual’s mother and father at her birth, the number of siblings, the level of physical and mental stress experienced at work, an index of deprivation for the individual’s current area of residence, history of disease in the family, information on the individual’s health behaviours which include smoking, sleep and diet (carbohydrate, protein, fat and calorie intake).

In this model, we controlled for a large range of potential cofounders. We included birth-year and department fixed effects, indicator variables for whether the individual lives with a spouse, achieved higher education, has been born preterm, lived on a farm for more than three months during childhood and whether the individual or her father belongs to a high socioprofessional category, the age of the individual’s mother and father at her birth, the number of siblings, the level of physical and mental stress experienced at work, an index of deprivation for the individual’s current area of residence, history of diseases running in the family and the variables concerning the individual’s health behaviour which include smoking, sleep and diet in terms of carbohydrate, protein, fat and calorie intake. Results are robust to the inclusion and exclusion of these different cofactors (tables are made available by the authors upon request), despite a large variation in the number of observations dropped due to the inclusion of certain cofactors with a large number of missing values. Notably, the results remain robust to the inclusion of health-affecting behaviours (tobacco consumption, sleep duration, and diet in terms of protein, lipid, carbohydrate and calorie intake), suggesting a direct association between early-life exposure and later-life risk for disease independently of potential changes in the health-affecting behaviours we observe in the data.

### Interactions with age

In our second set of models, we included age group interaction terms to see whether experiencing hunger at different ages had differential effects on later life health. For higher statistical power, we used the indicator variable equal to one if the individual suffered from any of the health conditions and zero otherwise. Column 1 in Table 3 shows a positive association between having suffered hunger during early life and the risk to suffer from any health condition (type 2 diabetes and/or hypertension) later in life, which increased by 2.83% (95% CI: 1.1%–4.6%).

**Table 3:** Effect of having experienced hunger on developing any health condition.

	Any condition	Any condition	Any condition	Any condition
Hunger	0.0283^***^		0.0306^***^	
	(0.0088)		(0.0094)	
Hunger x born after WWII		0.0245		−0.0100
		(0.2192)		(0.2094)
Hunger x born during WWII		0.0342^*^		0.0341^*^
		(0.0185)		(0.0195)
Hunger x born 1935-39		0.0367^**^		0.0365^**^
		(0.0148)		(0.0156)
Hunger x born 1930-34		0.0185		0.0241
		(0.0165)		(0.0179)
Hunger x born 1925-29		0.0172		0.0227
		(0.0237)		(0.0268)
Health behaviours	No	No	Yes	Yes
Num. obs.	30170	30170	26861	26861
Log Likelihood	−18536.20	−18535.70	−16341.97	−16341.76

***p < 0.01, **p < 0.05, *p < 0.1.

“Any condition” denotes a dichotomous variable equal to one if the individual was affected by any of the health conditions and zero otherwise. All models includes birth-year and department fixed effects, indicator variables for whether the individual lives with a spouse, achieved higher education, has been born preterm, lived on a farm for more than 3 months during childhood and whether the individual or her father is of high socioeconomic status, the age of the individual’s mother and father at her birth, the number of siblings, the level of physical and mental stress experienced at work, an index of deprivation for the individual’s current area of residence and history of disease in the family.

**Figure 1:**
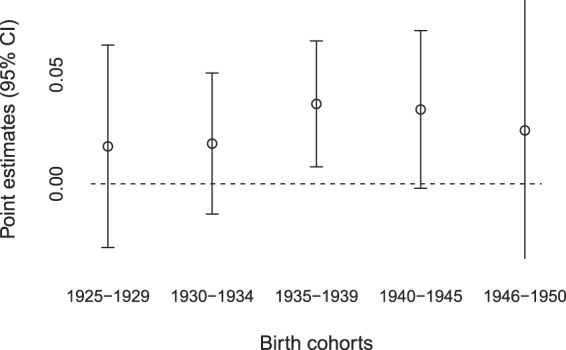
Effect of exposure to hunger on the probability of suffering from any health condition (type 2 diabetes and/or hypertension) at adulthood. The arrows show the 95% confidence interval. Only the results for individuals born from 1935 to 1939 are statistically significantly different from 0. The estimate for individuals born from 1946 to 1950 is not precisely estimated as few individuals declared having suffered from WWII-related hunger.

In column 2 of the same Table, we reported the estimates for the model with the age group interaction terms. The coefficients were of a higher magnitude for individuals who have been born from 1935 to 1939 (coefficient on “Hunger x born 1935-1939”) and were therefore aged 0 to 5 at the beginning of exposure (the onset of WWII). The association appeared to exist also for individuals born during the war (coefficient on “Hunger x born during WWII”) but the result is less statistically significant. Exposure to hunger does not yield statistically significant results for individuals who were older than 5 at the time of exposure. Figure 1 presents the point estimates and 95% confidence intervals graphically. Finding that the adverse effects of exposure to hunger were mainly present in individuals exposed before age 5 is suggestive evidence for the existence of a sensitive period of development during the first 5 years of life during which individuals appear to be particularly vulnerable to the exposure to adverse conditions.

The associations between exposure to hunger and later-life health remained when we adjusted for health-affecting behaviours (tobacco consumption, sleep duration, and diet in terms of protein, lipid, carbohydrate, and calorie intake). This can be seen from the coefficients presented in Columns 3 and 4 from models including health-affecting behaviours which are similar to the coefficients presented in Columns 1 and 2 from the models in which we do not adjust for health-affecting behaviours. This suggests a direct association between early-life exposure and later-life risk for disease independently of potential changes in the health-affecting behaviours we observe in the data.

### WWII and changes in health determinants

To investigate the potential indirect pathways through which early-life exposure to hunger could affect adult health, we examined whether having suffered from hunger was associated with changes in health determinants such as educational achievement and health-affecting behaviours including sleep duration, tobacco smoking, and diet in terms of carbohydrate, protein, lipid, and calorie intake. Results are reported in Table 4. The experience of hunger during childhood was positively correlated with tobacco consumption and inversely associated with average sleep duration and achieved educational level. We did not find evidence for associations with diet. We adjusted for a range of cofactors including indicator variables for whether the individual lived with a spouse, had been born preterm, had lived on a farm for more than three months during childhood, whether the individual or her father belonged to a high socioprofessional category, the age of the individual’s mother and father at her birth, the number of siblings, the level of physical and mental stress experienced at work, and an index of deprivation for the individual’s current area of residence. All models included birth-year and birth-place fixed effects.

**Table 4:** Effect of having experienced hunger on health determinants.

	Smoking	Sleep	Education
Hunger	0.0277^**^	−0.0836^***^	−0.0407^**^
	(0.0090)	(0.0210)	(0.0132)
Num. obs.	30145	26881	36378
Log Likelihood	−18461.8580		
R^2^		0.0092	0.1775

***p < 0.001, **p < 0.01, *p < 0.05.

All models are adjusted for birth-year and department fixed effects, indicator variables for whether the individual, has been born preterm, lived on a farm for more than 3 months during childhood and whether the individual or her father is of high socioeconomic status, the age of the individual’s mother and father at her birth, and the number of siblings.

## Discussion

We exploited data on 90,226 women from the French prospective cohort study E3N to estimate the long-run effects of exposure to World War II-related food deprivation during early life on the risk of developing type 2 diabetes, hypertension, and obesity at adulthood, while considering potential confounders. We also investigated whether the effects depended on the individual’s age at exposure. Finally, we examined the relationship between food-deprivation and subsequent health-affecting behaviours to explore potential indirect effects on health of severe food-deprivation.

We found that the experience of WWII-related food deprivation during early life was linked to a higher risk of developing type 2 diabetes (+0.7%, 95% CI: 0.073–1.37%) and hypertension (+2.6%, 95% CI: 0.81–4.45%) in adulthood. This still held true when we adjusted for a range of cofactors including health-affecting behaviours. The health of individuals who had been exposed up to age 5 appeared to be more affected than the health of individuals exposed at a later age. Exposure to hunger during early-life was also associated with different health determinants. We found a negative association between having suffered from hunger and educational achievement and mean sleep duration whereas the probability of ever smoking tobacco appeared to be higher among affected individuals.

Many studies investigated health outcomes in cohorts exposed to famine, and several found evidence that early malnutrition played a role in the origins of type 2 diabetes and high blood pressure. It was reported that women aged 6–8 at the peak of starvation during the 1941–44 Leningrad siege had higher systolic blood pressure compared to unexposed individuals born during the same period^11^. Women exposed to the 1944–1945 Dutch famine had an increased age-adjusted type 2 diabetes hazard ratio relative to unexposed women^29^. Severe under-nutrition at ages 11–14 among individuals exposed to the Dutch famine was reported to be associated with a higher probability of developing diabetes mellitus and/or peripheral arterial disease at ages 60–76^10^. Our findings are consistent with these results. Concerning the critical window of exposure, however, our findings differed from the aforementioned studies. We found that individuals exposed during early childhood (up to age 5) were more strongly affected whereas the previously cited studies identified mid- and late childhood as potential critical periods of exposure.

Relatively little information is available on the association between famine exposure and changes in health-affecting behaviours later in life. It was reported that early-life exposure to famine was associated with higher prevalence of smoking and physical activity later in life, but not with higher risk of unhealthy diet or of high alcohol consumption^30^. The negative association we identified between early-life hunger and sleep is in line with previous research indicating that disturbances of sleep are widespread in victims of adverse childhood experiences^31,32^. Our findings of a negative correlation between suffering from hunger and educational achievement are supported by Jyoti *et al*.^16^ who found that food insecurity negatively affects school children’s academic performance.

Within the Developmental Origins of Health and Disease (DOHaD) framework, the relationship between early life exposure and health trajectories across adulthood has been explained using both latency and pathway models. The latency model links early-life exposure to adult health outcomes in a direct manner independently of intervening life circumstances. It proposes that early-life exposures can program long-term or permanent changes in biological and behavioural systems^1,2^. This is based on the notion of biological imprinting which has been theorised by David Barker’s work^33,34^ who suggested that factors during early childhood make a biological imprint on the human organism in a way that makes it more susceptible to illness later in life. For example, Hertzman^1^ hypothesised that in utero and early life circumstances could affect the structure and functioning of the central nervous system, which interacts with the immune, hormonal, and clotting systems to increase or decrease susceptibility to disease.

In contrast, the pathway model proposes that early-life exposure indirectly relates to adult health outcomes through effects on health determinants and health-related behaviours. Ben-Shlomo^35^ discussed the pathway model in terms of “chains of risk” where one adverse exposure conditions the individuals’ response and leads to other adverse events. Negative childhood experiences may lead to unhealthy behaviours and poor school performance in adolescence, and limited opportunities in adulthood. Inadequate resources and stressful life circumstances in adulthood, in turn, increase the risk of morbidity and mortality. This pathway model is closely linked to the notion of allostatic load^35^, a concept used to explain how experiences become biologically embedded to influence health. The healthy functioning of a body requires ongoing adjustment through fluctuations in the physiologic system to respond to stressors. This process is referred to as allostasis^36^ and involves the sympathetic nervous system, the neuroendocrine system (in particular the hypothalamic-pituitary-adrenal axis), and the immune system. Internal needs are temporarily subjugated to external ones^37^, which is essential for survival but may have negative long-term effects, promoting illness in the case of individuals exposed to multiple acute or chronic stressors^38^. Multiple recurring stressors leave a physiologic stamp on the body^39^, which is reflected in bio-markers and in derangement of the body systems they have affected. This physiological stamp is the allostatic load, and it impairs the body’s ability to adapt to future stressors^40^.

The latency and pathway models are not competing explanations, but are thought to be intertwined in a complex manner. Chronic disease may be the long-term outcome of childhood conditions and experiences combined with cumulative exposures across adulthood^41^. Susceptibility to diseases is embedded in individuals’ biological makeup, but diseases are expressed and maintained in particular social, economic, and cultural environments^2,42,43^. Our findings are compatible with both the predictions of the latency and pathway models. We found a direct association between early-life exposure and later-life risk of diabetes and hypertension, which appears to persist when adjusting for health determinants recorded in E3N (i.e. education and health-affecting behaviours). We also found that exposure was linked to differences in these health determinants which, in turn, are likely to influence health^18,44,45^.

We investigated associations between early-life exposure to hunger and later life risk of diabetes and hypertension using verified data on health as opposed to self-declared data which is most commonly used in the literature. Our results are therefore not subject to potential bias from misreported health. The richness of the E3N data allowed us to adjust for a large range of family and individual characteristics including, most importantly, socioeconomic status which has been shown to be an important determinant of adult health outcomes^46^. We have also been able to explore the possibility that early-life food deprivation affected health determinants such as educational achievement, and health-affecting behaviours including sleeping duration, diet, and tobacco smoking, which may mediate the effects on health. In the existing literature, relatively little information is available on the association between famine exposure and changes in health determinants and behaviours later in life.

Our study has certain limitations. Despite having adjusted the regression models for a wide range of cofactors, we cannot conclude that the effects we find are causal. Exposure to hunger may still be correlated with unobserved individual and household characteristics which may also drive health determinants and/or health outcomes and may therefore bias our estimates. We also note that individuals who suffered from food deprivation could have been exposed to other WII-related stressors. We were not able to control for exposure to such stressors and their potential effects on health determinants and health outcomes. Finally, many potentially important health determinants are absent from the E3N data although they could have been affected by exposure to hunger and have influenced health outcomes. Our findings of a direct association between early-life exposure and adult health could be due to not being able to adjust for important unobserved factors which may have mediated the health outcomes. Finally, the estimates in our study are likely to be a lower bound for the effects of exposure to hunger on health outcomes as there may have been a change in the composition of the population caused by differential mortality. If the least healthy are more likely to die, the pool of survivors may on average be of better health. In such a case, the average health in a population that was intensely affected by hunger could be better than the average health of a less affected population, leading us to underestimate the impact of exposure to hunger on health.

To conclude, we briefly summarise our findings and provide policy recommendations as follows. We found that early-life exposure to food deprivation during childhood and especially during the first five years of life increased the risk of suffering from hypertension and type 2 diabetes at adulthood. This still held true when we adjusted for health determinants and health-related behaviours at adulthood. Exposure was also associated with differences in health determinants and behaviours, such as lower educational achievement, lesser amount of sleep, and higher probability of ever smoking tobacco at adulthood. Our findings are compatible with both the latency and the pathway models proposed in the Developmental Origins of Health and Disease framework which theorise the existence of both a direct link between early-life exposure and adult health outcomes due to the programming of long-term or permanent changes in biological systems, and an indirect link where early-life exposure affects health determinants such as health-related behaviours which, in turn, impact health outcomes. This new empirical evidence for the predictions of the Developmental Origins of Health and Disease hypothesis is potentially useful information for the targeting of policy interventions to improve the health outcomes of vulnerable individuals. Our results suggest that it is reasonable for policy makers to shift away from an emphasis on treatment in the later stages of disease to earlier, more effective preventive strategies and interventions to maximise health development, for example by increasing investments in early-childhood prevention programmes. In addition, policy-makers could target their policies at improving health-affecting behaviours (e.g. smoking, sleep hygiene), as health effects appear to be partially mediated by changes in these behaviours.

## Supplementary information

Supplementary Information.
