# Supplementary figures and images for: Associations between early-life food deprivation during World War II and risk of hypertension and type 2 diabetes at adulthood
Source: Sci Rep. 2020 Apr 1;10:5741. doi: 10.1038/s41598-020-62576-w (PMC7113250; doi:10.1038/s41598-020-62576-w)

Point estimates (95% CI)

0.05  
0.00

1925–1929

1930–1934

1935–1939

1940–1945

1946–1950

Birth cohorts

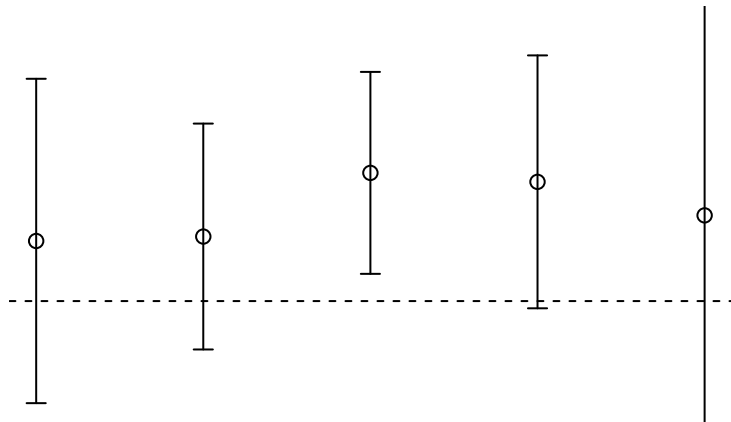

Supplement: Supplementary file 1 — Supplementary Information. [file 41598_2020_62576_MOESM1_ESM.pdf]
